# A Multidisciplinary Team Approach Is Highly Effective in the Management of Nondiagnostic Bone Tumour Biopsies: A 10-Year Retrospective Review at a Specialist Sarcoma Unit

**DOI:** 10.1155/2022/7700365

**Published:** 2022-03-28

**Authors:** Khabab Osman, Shakir Hussain, Frederick Downes, Vaiyapuri Sumathi, Rajesh Botchu, Scott Evans

**Affiliations:** ^1^The Royal Orthopaedic Hospital, Birmingham B31 2AP, UK; ^2^Birmingham Orthopaedic Network (BON), The Knowledge Hub, B31 2AP, Birmingham, UK

## Abstract

Nondiagnostic (ND) biopsies are frequently encountered during the investigation of bone tumours and can lead to treatment delay. We performed a retrospective review of all ND bone tumour biopsies discussed at our regional MDT meeting between 2004 and 2014 with the aim of establishing the incidence of ND biopsies, identifying any factors that could predict the requirement for repeat biopsies, and evaluating the effectiveness of multidisciplinary team (MDT) decisions. We identified 98 ND out of 4949 biopsies. Diagnostic yield (DY) was 98%, 76%, and 40% for the first, second, and third successive biopsy, respectively. With an MDT approach utilising radiological and clinical information, the diagnostic success rate achieved was 99%, 85%, and 80% for the first, second, and third biopsies, respectively. Although a repeat biopsy was only performed in 34% of cases, there were no patients originally diagnosed with a benign lesion that re-presented with the same lesion subsequently being malignant throughout the study period. Malignant primary bone tumours (*p* < 0.01) and malignant secondary tumours (*p*=0.02) were more likely to undergo repeat biopsy compared to benign and infective lesions. Upper limb (*p*=0.04) and lower limb (*p*=0.03) were more likely than pelvic and spinal tumours to undergo a repeat biopsy. Tumours of haematological origin frequently required multiple biopsies. Our study demonstrated that a specialist MDT approach leads to high diagnostic rates and is a safe and effective method of preventing unnecessary, repeat biopsies where the initial biopsy is ND.

## 1. Introduction

Malignant primary bone tumours (mPBT) are rare and account for only 0.2% of all neoplasms. Although laboratory and radiological investigations can generate potential differential diagnoses, a tissue biopsy is usually needed to provide a definitive diagnosis. In the UK, national guidelines published in 2010 and updated in 2016 suggest that the investigation and treatment of all suspected mPBT be conducted at specialist bone tumour centres and guided by a multidisciplinary team (MDT). [1] Failure of this can lead to misdiagnosis, initiation of incorrect treatment, repeat biopsies, unnecessary surgery, and recurrence. [2].

Image-guided percutaneous core needle biopsy (pCNB) is now an established method of acquiring histopathology bone specimens and has been shown to be as good as open biopsy in terms of diagnostic rate and ability to accurately distinguish malignant from benign tumours, low-grade from high-grade tumours, and specific diagnosis [3, 4]. Owing to the minimally invasive nature of pCNB, there may be a reduced risk of biopsy tract seeding, local recurrence, and overall complication rate (less than 1%) making it a safer alternative to open biopsy [3–5].

On occasion, a diagnosis cannot be made from the tissue sample taken, thereby yielding a “nondiagnostic (ND) biopsy.” ND biopsies can be seen for multiple reasons: normal tissue obtained erroneously rather than from the target site, insufficient material obtained for complete analysis particularly associated with underlying fracture, a sufficient tissue sample that can be analysed but cannot be categorised into a known histological class, or necrotic tissue or cyst content only. Certain tumours are reported to be associated with a low DY including LCH and malignant small round blue cell tumours while tumours that are large, lytic lesions or possess an extraosseous soft tissue component may have a higher DY [6–8]. An ND biopsy may be disconcerting for a patient awaiting a diagnosis and can lead to a delay in treatment, possibly resulting in a poorer outcome.

There are few studies describing the management of ND biopsies of suspected malignant bone tumours with small numbers (*n* = 15 and *n* = 26) [8, 9]. The aim of our study was to investigate the “clinical utility” of ND bone biopsies and assess what factors may influence the likelihood of obtaining a ND repeat biopsy in patients being investigated for malignant bone tumours.

## 2. Methods

We retrospectively identified all ND biopsies discussed at our sarcoma MDT between 2004 and 2014 from the institution's prospectively maintained database, the vast majority of which were first discussed prior to biopsy. We allowed a latency period of 5 years between biopsy and data collection to identify any cases presenting late with missed malignant tumours.

We collected data on patient age at the time of biopsy, anatomical site, biopsy type, and tumour type (as per the final MDT diagnosis). Anatomical sites were grouped into upper limb (UL), lower limb (LL), pelvis, and spine. Biopsy types were grouped into pCNB (CT, US, and XR guided), open biopsies (open CNB, incisional, intralesional curettage, and reamings), and surgical excision biopsy. Tumour types were categorised into malignant primary bone tumour (mPBT), malignant secondary bone tumour (mSBT), benign lesion (BL), and lesions secondary to infection. mSBT included metastatic carcinomas (Ca) and tumours of haematological origin. Where available, resection specimens were used to corroborate the final diagnosis.

We recorded whether each biopsy was ND, led to a “tissue diagnosis, or led to a “clinical diagnosis” despite the lack of histological confirmation. We also recorded the total number of biopsies required to make a diagnosis leading to a definitive management plan.

Diagnostic yield (DY) was defined as the percentage success rate of a biopsy achieving a “tissue diagnosis,” which included biopsies that effectively exclude malignancy. Where the MDT made a diagnosis based on available clinical, radiological, and laboratory information without histopathology confirmation, this is termed as “clinical diagnoses.” Thereby, “diagnostic success rate” (DSR) is defined as the combined rate of “tissue diagnosis” and “clinical diagnoses.”

### 2.1. Statistical Analysis

Analysis was performed using IBM SPSS version 26. Multinomial logistic regression with a “stepwise backward elimination” method was used to examine the relationship between multiple factors and the likelihood of requiring a repeat biopsy. Ordinal logistic regression was used to examine the relationship with increasing number of biopsies required for a diagnosis. Direct comparisons between categorical variables were performed using Pearson's chi-square test. Significance was considered as *p* < 0.05.

## 3. Results

**Figure 1:**
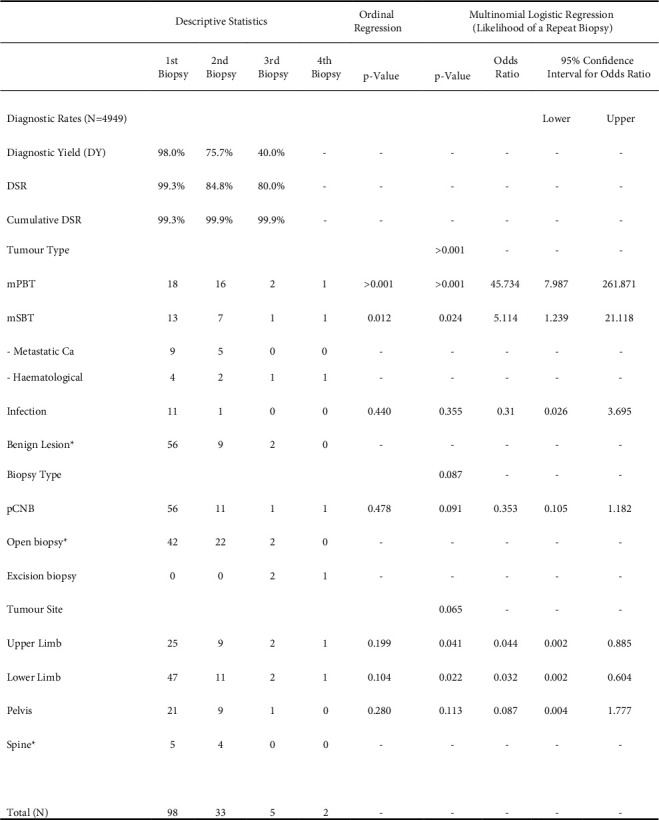
Results table: descriptive statistics including diagnostic rates and frequencies of biopsies grouped by tumour type, biopsy type, and tumours site for successive biopsies. The results of ordinal and multinomial logistic regression are shown on the right-hand columns. *p* values for groups represent the “main effect” of that group, and individual factors are represented with odds ratios, confidence intervals, and *p* values in relation to the reference category (^*∗*^). Pearson's chi-square for “goodness of fit” was 80.0 (*p*=0.51) and 84.7 (*p*=0.083) for the multinomial regression and ordinal regression models, respectively. DSR = diagnostic success rate of the MDT, mPBT = malignant primary bone tumour, mSBT = malignant secondary bone tumour, and pCNB = percutaneous core needle biopsy.

98 patients with ND biopsies were identified from a total of 4949 biopsies.  Figure 1 shows the complete results of the study.

### 3.1. Diagnostic Rates

The DY, DSR, and cumulative DSR for each successive biopsy are shown in Figure 1. The DY was 98%, 76%, and 40% at the first, second, and third biopsies, respectively. DSR was consistently higher but reduced in similar trend with each successive biopsy from 99%, 85%, and 80% at the first, second, and third biopsies, respectively.

Despite a ND histology report for the first biopsy, no further biopsy was undertaken for 66% of cases (*n* = 65). In these cases, the biopsy did not reveal evidence of suspicious cells and, in combination with clinical and radiological features, malignancy was excluded by MDT discussion. This is with the exception of 1 patient who died before a planned repeat biopsy. The difference between DY and DSR is accounted for by such cases.

Although we could not assess the effects of various factors on the DY of the first biopsy (*N* = 4949), we did not find any significant effect of age, tumour site, biopsy type, or tumour type on the likelihood of a tissue diagnosis for the second biopsy (*n* = 33).

### 3.2. Patient Age

The mean age at the time of the first biopsy was 39 years (range 5 to 89). There was no statistically significant association between patient age and the likelihood of undergoing a repeat biopsy or increasing number of biopsies. Increasing patient age was however correlated with a significantly higher likelihood of being diagnosed with mPBT (*p*=0.02), mSBT (*p* < 0.01), and an infected lesion (*p*=0.02) when compared to BL.

### 3.3. Tumour Type

**Figure 2:**
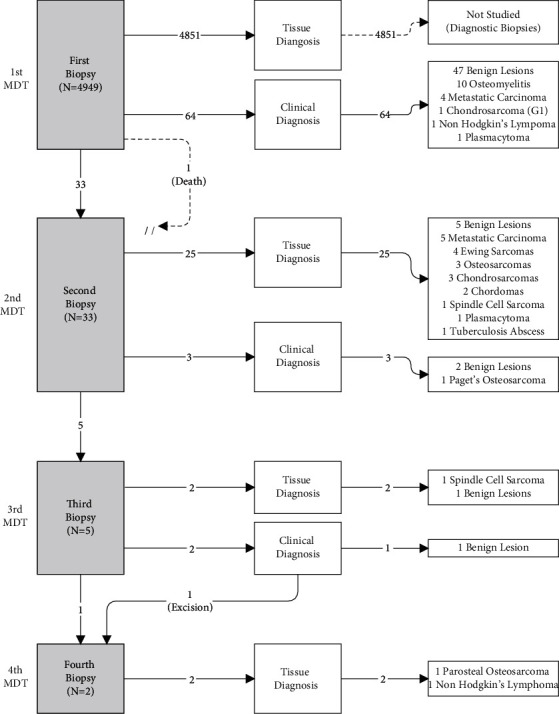
ND biopsy diagnosis flowchart showing how each diagnosis was reached through consecutive MDT discussions. Cases where the first biopsy was diagnostic were not studied, and therefore, no specific diagnoses are listed. All numbers represent frequencies.

Tumour type significantly influenced the likelihood of having a repeat biopsy (*p* < 0.001).  Figure 2 shows how each diagnosis was reached. The results for each category of tumour type are presented separately. As far as we are aware, no patients re-presented to our unit with malignant tumours that were previously diagnosed as benign (zero false negatives).

  mPBT: when compared to benign lesions, mPBT were significantly more likely to undergo a repeat biopsy after an initial ND biopsy (*p* < 0.01) and were positively correlated with an increasing number of biopsies (*p* < 0.01). Our study included 18 mPBT, of which 16 had a repeat biopsy yielding a diagnosis in 81% of cases (*n* = 13). Of note, one chondrosarcoma was diagnosed based on clinical and radiological features and underwent surgical excision without the need for a repeat biopsy and a Paget's osteosarcoma with lung metastasis at presentation treated palliatively despite an inconclusive open repeat biopsy. 2 patients had a total of 3 biopsies, including an atypical parosteal osteosarcoma initially mistaken as a soft tissue tumour and a suspected recurrence of Grade II chondrosarcoma in a hindquarter amputation stump found to be high-grade dedifferentiated spindle cell sarcoma following excision.
  mSBT: when compared to benign lesions, mSBT were also significantly more likely to undergo a repeat biopsy (*p*=0.02) and an increasing number of biopsies (*p*=0.01). All 9 cases of metastatic Ca were diagnosed within a maximum of 2 biopsies. A clinical diagnosis was given for 4 out of the 9 cases without a repeat biopsy. The remaining 5 cases underwent a single repeat biopsy, which yielded tissue diagnoses. Tumours of haematological origin (*n* = 4) included 2 plasmacytomas and 2 non-Hodgkin's (NH) lymphoma, of which 2 underwent a repeat biopsy and 1 lymphoma patient required a total of 4 biopsies before diagnosis.
  Benign lesions: in total, 56 out of the 98 ND biopsies were diagnosed as benign lesions. A biopsy was repeated in 9 cases (16) of which 5 were confirmed benign on histology, 2 were clinically diagnosed as benign lesions, and 2 required a third biopsy (an atypical haemangioma and a persistently painful osteoid osteoma after radiofrequency ablation).
  Infection: 11 out of 98 ND biopsies were lesions secondary to infection. The MDT made a clinical diagnosis in 10 out of the 11 (91%) at the first meeting, and only 1 patient underwent a repeat biopsy, yielding a tissue diagnosis of tuberculosis abscess.

### 3.4. Biopsy Type

**Figure 3:**
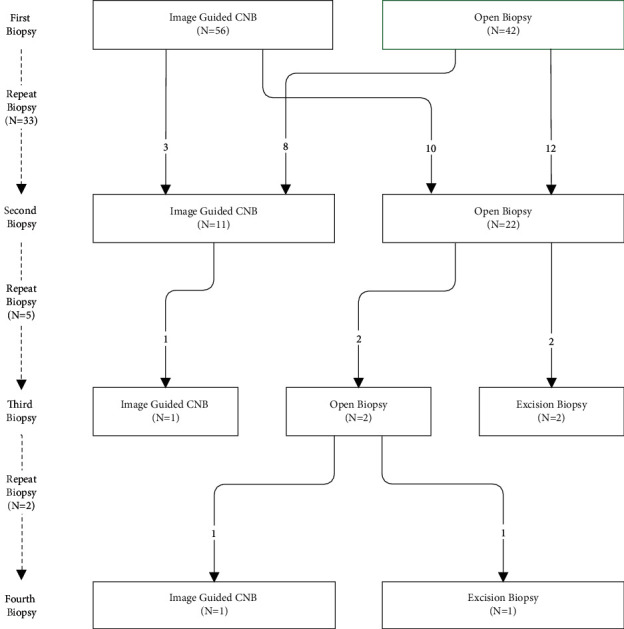
Biopsy type flowchart showing the biopsy technique used at each consecutive biopsy. The arrows indicate the number of patients moving from one biopsy type in the former row to another biopsy type in the latter row.

Figure 3 illustrates the frequency of each type of biopsy performed and the frequency of changes in technique when a biopsy is repeated.

We found no significant relationship between biopsy type and the likelihood of undergoing a repeat biopsy, but pCNB was significantly more likely to lead to an MDT clinical diagnosis (chi-square = 4.19, *p*=0.02). However, the utilisation of pCNB decreased with each successive biopsy from 57% (*n* = 56), 33% (*n* = 11), and 20% (*n* = 1) at the first, second, and third biopsies, respectively. Where biopsies were repeated, biopsy type was changed in 18 out of 33 cases and was successful in achieving a diagnosis in 78% of these. pCNB was changed to an open biopsy in 10 out of 13 cases (77%). All 3 repeat pCNB successfully confirmed benign lesions. Open biopsies were repeated in 12 out of 20 cases (60%). The remaining 8 were changed to pCNB, all of which successfully confirmed malignant tumours.

### 3.5. Tumour Site

UL and LL tumours were significantly less likely than spinal tumours to undergo a repeat biopsy (*p*=0.04 and *p*=0.02, respectively). 4 out of 5 (80%) spinal tumours underwent a repeat biopsy in contrast to 12 out of 25 (48%) upper limb, 12 out of 37 (32%) lower limb, and 10 out of 21 (38%) pelvic tumours. No significant association between tumour site and histological tumour type was identified.

## 4. Discussion

It is well established that improved outcomes are seen when the investigation and management of suspected sarcoma is directed by dedicated MDTs in specialist centres [2]. We have previously shown that daily MDT meetings in our unit have helped to reduce time to diagnosis and decrease overall patient travel and facilitate communication with patients and referring clinicians [10]. Our MDT includes orthopaedic surgeons, radiologists, oncologists, and histopathologists, all specialising in musculoskeletal oncology who come together to decide the most appropriate biopsy technique, specific site of biopsy, sample preparation, and histological analysis techniques on a case-by-case basis. When faced with an ND biopsy result, the MDT engages in evidence-based discussion to determine whether further investigation or a repeat biopsy is required for suspicious bone lesions. As far as we are aware, this is the largest series of ND bone biopsies (*n* = 98), with previous studies having a maximum of 26 ND biopsies [8, 9].

Diagnostic Yield (DY) refers to the percentage of biopsies leading to a histopathological tissue diagnosis. Previous studies that focused predominantly on pCNB have reported DY ranging from 67% to 96% (combined *N* = 1389) [3, 4, 6, 8, 11–15]. In our study, which included 4849 biopsies of different types co-ordinated by an MDT, we demonstrated a superior DY of 98%. Since DY does not differ for pCNB compared to open biopsies [4], the high DY cannot be attributed to the presence of open biopsies in our study. Although on-site frozen section and imprint cytology were not available at our institution, it has been shown to be useful in helping establish the adequacy of biopsy specimens and could further improve the DY, especially for repeat biopsies [16]. Despite the lack of such techniques, our superior diagnostic rates and lack of false-negative diagnoses (initially labelled benign tumours re-presenting as malignant) can likely be attributed to the ‘bespoke' specialist MDT approach compared to studies where a standardised pathway was employed.

The MDT diagnosed 66% of cases (*n* = 65) based on the available clinical, radiology, and pathology information without deferring to a repeat biopsy, leading to a 99.3% DSR for the first biopsy. This confirms the findings of Didolker et al. and Omura et al. demonstrating that ND biopsies can be useful for decision-making and that a clinical and radiological diagnosis is possible in many cases [11, 14]. Similar to Wu et al., our data demonstrate that the DY and DSR reduce with each successive repeat biopsy [9], falling from 98% and 99.3% at the first biopsy to 40% and 80% at the third biopsy, respectively. Therefore, the necessity of repeat biopsies should be carefully considered by the MDT and can often be safely avoided. The improved DSR relative to DY highlights the importance of considering the clinical history, blood tests, and radiological investigations in discussion rather than relying on tissue diagnosis alone to distinguish benign and malignant tumours.

Malignant tumours represented 32% of our cohort of ND biopsies. As expected, we demonstrated mPBT and mSBT were more likely to undergo a repeat biopsy (*p* < 0.01 and *p*=0.02, respectively) and an increasing number of biopsies (*p* < 0.01 and *p* < 0.01, respectively) when compared to benign lesions. This likely reflects the ability of the MDT to exclude malignancy based on clinical and radiological information, as was the case for 47 out of 56 benign lesions. Benign lesions are known to be less likely to yield a tissue diagnosis and can be difficult to differentiate from low-grade sarcoma, [6, 11, 13, 14], and therefore, a repeat biopsy may be less useful. Conversely, a tissue diagnosis for mPBT is vitally important and has huge implications for patient management consisting of multimodal therapy, including chemotherapy, radiotherapy, and invasive orthopaedic oncological surgery. In such cases, a repeat biopsy is vital and was performed for 17 out of 18 cases eventually diagnosed as mPBT, with the exception of a recurrent chondrosarcoma. However, there are instances of failed repeat biopsies for strongly suspected malignant tumours where it may be necessary to proceed with surgical excision without confirmatory histology following detailed MDT discussion and agreement of the patient. This occurred for 3 tumours in our cohort, of which 2 were mPBT and 1 was an atypical haemangioma.

Similar to other studies, tumours of haematological origin (lymphoma and myeloma) frequently required repeat biopsies in our study [4, 6, 7, 15, 17, 18], possibly due to intralesional architectural variability, susceptibility to crush artefact, and the presence of satellite lesions which may be missed. In contrast, the MDT was frequently able to make a clinical diagnosis for metastatic carcinomas (4 out of 9) and repeat biopsies, when performed, were successful in all instances. Similarly, infective lesions were readily diagnosed without a repeat biopsy (10 out of 11 lesions).

MDT discussion is key to deciding the optimum biopsy method. CT-guided pCNB remains our preferred method of acquiring bone biopsy specimens, except for highly sclerotic tumours which are less likely to yield useful samples, tumours that are closely related to vascular or neurological structures and open biopsy is safer, or where tissue diagnosis is predicted to be difficult, and open biopsy would harvest more tissue for analysis [7, 8, 13]. These factors likely account for the high utilisation of open biopsies in this study (42 out of 98) and are further illustrated by the MDTs' clear preference for an open technique when a repeat biopsy is required. However, we found that nondiagnostic pCNB was more likely to lead to a clinical diagnosis than open biopsies (*p*=0.02), which we attribute to the ability of the radiologist to review the needle position during the MDT meeting and determine whether the tumour epicentre may have been missed. Where nonmalignant tissue is yielded from an optimally placed needle in a lesion that is likely to be benign, malignancy can be confidently excluded.

In agreement with Hua et al. [17], we found that upper and lower limb tumours were significantly less likely to undergo a repeat biopsy compared to spinal tumours (*p*=0.04 and *p*=0.02, respectively), possibly due to anatomical constraints causing difficulty acquiring suitable biopsy specimens. On such occasions, an open biopsy may be preferred.

### 4.1. Limitations

Our prospectively collected database is subjected to the expected problems of inaccurate data entries, missing information, and inconsistent terminology. Although our specialist pathologists reviewed all specimens, some initial biopsies were performed at the referring hospitals prior to MDT discussion. We were unable to account for any missed false negatives, recurrences, or unexpected deaths that may have presented to other hospitals and were not referred back to our MDT.

Some suspected benign lesions undergo therapeutic procedures such as radiofrequency ablation and intralesional curettage where “opportunistic biopsies” are taken. Where there was no prior definitive diagnosis, they were considered repeat biopsies, which may lead to an overestimation of the frequency of repeat biopsies for benign lesions.

## 5. Conclusions

Our study corroborates the UK national guidelines and adds credence that a specialist MDT approach is vital in the investigation of bone tumours. Using an MDT approach, a DY of 98% and DSR of 99.2% can be achieved without any missed malignancies. When dealing with ND biopsies, appropriate management plans can be made in the majority of cases without a repeat biopsy. The predictors of ND biopsy are complex and multifactorial; a specialist multidisciplinary team can identify cases that may yield repeat ND biopsy results and select the appropriate strategy to improve the DY and thereby prevent treatment delay.

## Data Availability

Data are contained in an SPSS data file (.sav) and can be provided on request by contacting the corresponding author.

## References

[1] Gerrand C., Athanasou N, Athanasou N. (2016). UK guidelines for the management of bone sarcomas. *Clinical Sarcoma Research*.

[2] Mankin H. J., Mankin C. J., Simon M. A. (1996). The hazards of the biopsy, revisited. For the members of the musculoskeletal tumor society. *The Journal of Bone & Joint Surgery*.

[3] Dupuy D. E., Rosenberg A. E., Punyaratabandhu T., Tan M. H., Mankin H. J. (1998). Accuracy of CT-guided needle biopsy of musculoskeletal neoplasms. *American Journal of Roentgenology*.

[4] Kiatisevi P., Thanakit V., Sukunthanak B., Boonthatip M., Bumrungchart S., Witoonchart K. (2013). Computed tomography-guided core needle biopsy versus incisional biopsy in diagnosing musculoskeletal lesions. *Journal of Orthopaedic Surgery*.

[5] Barrientos-Ruiz I., Ortiz-Cruz E. J., Serrano-Montilla J., Bernabeu-Taboada D., Pozo-Kreilinger J. J. (2017). Are biopsy tracts a concern for seeding and local recurrence in sarcomas?. *Clinical Orthopaedics & Related Research*.

[6] Nouh M. R., Abu Shady H. M. (2014). Initial CT-guided needle biopsy of extremity skeletal lesions: diagnostic performance and experience of a tertiary musculoskeletal center. *European Journal of Radiology*.

[7] Li Y., Du Y., Luo T. Y. (2014). Factors influencing diagnostic yield of CT-guided percutaneous core needle biopsy for bone lesions. *Clinical Radiology*.

[8] Wu J. S., Goldsmith J. D., Horwich P. J., Shetty S. K., Hochman M. G. (2008). Bone and soft-tissue lesions: what factors affect diagnostic yield of image-guided core-needle biopsy?. *Radiology*.

[9] Wu J. S., McMahon C. J., Lozano-Calderon S., Kung J. W. (2017). Journal club: utility of repeat core needle biopsy of musculoskeletal lesions with initially nondiagnostic findings. *American Journal of Roentgenology*.

[10] Hartley L. J., Evans S., Davies M. A., Kelly S., Gregory J. J. (2021). A daily diagnostic multidisciplinary meeting to reduce time to definitive diagnosis in the context of primary bone and soft tissue sarcoma. *Journal of Multidisciplinary Healthcare*.

[11] Didolkar M. M., Anderson M. E., Hochman M. G. (2013). Image guided core needle biopsy of musculoskeletal lesions: are nondiagnostic results clinically useful?. *Clinical Orthopaedics & Related Research*.

[12] Issakov J., Flusser G., Kollender Y., Merimsky O., Lifschitz-Mercer B., Meller I. (2003). Computed tomography-guided core needle biopsy for bone and soft tissue tumors. *The Israel Medical Association Journal: The Israel Medical Association Journal*.

[13] Mitsuyoshi G., Naito N., Kawai A. (2006). Accurate diagnosis of musculoskeletal lesions by core needle biopsy. *Journal of Surgical Oncology*.

[14] Omura M. C., Motamedi K., UyBico S., Nelson S. D., Seeger L. L. (2011). Revisiting CT-guided percutaneous core needle biopsy of musculoskeletal lesions: contributors to biopsy success. *American Journal of Roentgenology*.

[15] Yang J., Frassica F. J., Fayad L., Clark D. P., Weber K. L. (2010). Analysis of nondiagnostic results after image-guided needle biopsies of musculoskeletal lesions. *Clinical Orthopaedics & Related Research*.

[16] Kubik M. J., Bovbel A., Goli H., Saremian J., Siddiqi A., Masood S. (2015). Diagnostic value and accuracy of imprint cytology evaluation during image-guided core needle biopsies: review of our experience at a large academic center. *Diagnostic Cytopathology*.

[17] Hau M., Kim J., Kattapuram S. (2002). Accuracy of CT-guided biopsies in 359 patients with musculoskeletal lesions. *Skeletal Radiology*.

[18] Tsukushi S., Nishida Y., Yamada Y., Yoshida M., Ishiguro N. (2010). CT-guided needle biopsy for musculoskeletal lesions. *Archives of Orthopaedic and Trauma Surgery*.

